# Geschlechtersensible Prävention und Gesundheitsförderung für Kinder: Chancen und Herausforderungen bei der Umsetzbarkeit

**DOI:** 10.1007/s11553-022-01006-3

**Published:** 2022-12-29

**Authors:** Katharina Kreffter, Stefanie Wessely, Thuy Ha Nguyen, Stefanie Lisak-Wahl, Christine Joisten, Simone Weyers

**Affiliations:** 1 Hochschule Hamm-Lippstadt, University of Applied Sciences, Marker Allee 76–78, 59063 Hamm, Deutschland; 2 Abteilung Bewegungs- und Gesundheitsförderung, Institut für Bewegungs- und Neurowissenschaft, Deutsche Sporthochschule Köln, Am Sportpark Müngersdorf 6, 50933 Köln, Deutschland; 3 IGES Institut, Friedrichstr. 180, 10117 Berlin, Deutschland; 4 Akademie für Akademie für Öffentliches Gesundheitswesen in Düsseldorf, Kanzlerstr. 4, 40472 Düsseldorf, Deutschland; 5 HHU Medizinische Fakultät, Universitätsklinikum Düsseldorf, Centre for Health and Society, Institut für Medizinische Soziologie, Heinrich-Heine-Universität Düsseldorf, Moorenstr. 5, 40225 Düsseldorf, Deutschland

**Keywords:** Geschlechtersensibilität, Gender, Kindliche Entwicklung, Prävention und Gesundheitsförderung, Gender sensitivity, Gender, Child development, Prevention and health promotion

## Abstract

**Hintergrund:**

Bei Prävention und Gesundheitsförderung sollten Geschlechterunterschiede bereits im Kindesalter berücksichtigt werden. Allerdings fehlen hierzu sowohl theoretisch fundierte Konzepte als auch insbesondere praktische Handlungsempfehlungen.

**Ziel der Arbeit:**

Daher sollte ermittelt werden, was Wissenschaftler/innen und Praktiker/innen empfehlen, um Anbietende bei der Entwicklung geschlechtersensibler präventiver und gesundheitsförderlicher Maßnahmen für Kinder zu unterstützen.

**Material und Methoden:**

Es wurde ein mehrschrittiges qualitatives Vorgehen gewählt, um die Erfahrungs- und Wissensbestände aus Wissenschaft und Praxis zu erfassen. Das Vorgehen bestand aus einem Expertenworkshop mit Wissenschaftler/innen (*n* = 12) angelehnt an die nominale Gruppentechnik, sechs Fokusgruppen mit Fachkräften (*n* = 7; 6; 8; 6; 8; 4) und einem abschließenden Online-Workshop mit beiden Gruppen (*n* = 12).

**Ergebnisse:**

Die resultierenden Handlungsempfehlungen beinhalten die folgenden Punkte: Geschlecht und Lebenslage adressieren, Geschlechterrollen und Lebensweisen kennenlernen, Partizipation und Qualitätssicherung, Dokumentation von Praxisprojekten, Diversität in professionellen Teams, Geschlechtersensibilität in Qualifikationsmaßnahmen, Austausch und Vernetzung.

**Schlussfolgerung:**

Bei der Umsetzung von geschlechtersensibler Prävention und Gesundheitsförderung bei Kindern sind sowohl politische als auch gesellschaftliche und individuelle Ebenen zu berücksichtigen. Während das Thema in der praktischen Arbeit schon Beachtung findet und umgesetzt wird, besteht Forschungsbedarf insbesondere hinsichtlich konzeptioneller Entwicklung und Evaluationen.

**Zusatzmaterial online:**

Zusätzliche Informationen sind in der Online-Version dieses Artikels (10.1007/s11553-022-01006-3) enthalten.

## Abstract (en)

**Background:**

In prevention and health promotion, gender differences should be considered already in childhood. However, there is a lack of both theoretically well-founded concepts and, in particular, practical recommendations.

**Objectives:**

The aim of this study was to identify what researchers and practitioners recommend to providers in the development of gender-sensitive preventive and health promotion programs for children.

**Materials and methods:**

A multistep qualitative approach was chosen to capture the experience and knowledge of experts from both science and practice. It consisted of an expert workshop (*n* = 12) adapted from the nominal group technique, six focus groups with professionals (*n* = 7; 6; 8; 6; 8; 4), and a final online workshop with both groups (*n* = 12).

**Results:**

The resulting recommendations include the following: addressing gender and social circumstances; reflection of gender roles and lifestyles; participation and quality management; documentation of practice projects; diversity in professional teams; gender sensitivity in qualification measures; dialogues and networking.

**Conclusions:**

When implementing gender-sensitive prevention and health promotion for children, political, societal, and individual aspects should be considered. While the topic already receives attention and is implemented in practical work, there is a need for research, especially with regard to conceptual development and evaluation.

## Hintergrund und Fragestellung

Jungen und Mädchen unterscheiden sich bereits vor Eintreten von Adoleszenz und Pubertät deutlich in Gesundheit, Gesundheitsverhalten und Entwicklung [14, 22, 43, 46]. Präventive und gesundheitsfördernde Maßnahmen sollten daher Geschlechterunterschiede schon für die spezifische Altersgruppe der Kinder berücksichtigen. Dies entspricht der allgemeinen Forderung nach geschlechtergerechten oder geschlechtersensiblen Interventionen [8, 26, 42]. Geschlechtersensible Gesundheitsförderung und Prävention haben das Ziel, geschlechtsbedingte Ungleichheiten von Gesundheitschancen zu reduzieren [7]. Doch wie sollen Projektträger/innen in den verschiedenen Settings dies in der Arbeit mit Kindern praktisch umsetzen?

In der Literatur finden sich einige allgemeine konzeptionelle Überlegungen zur geschlechtersensiblen Prävention und Gesundheitsförderung wie beispielsweise: Berücksichtigung der Kategorie Geschlecht bei der Maßnahmenplanung und -umsetzung auf verschiedenen Ebenen [24], geschlechtergerechte Operationalisierung von Qualitätsparametern [44], spezifische Probleme und Handlungsfelder bei Männern und Jungen [2] und multisektorale Herangehensweise [42]. Diesen Ansätzen ist allerdings gemein, dass sie nicht primär auf die Altersgruppe der Kinder fokussieren.

Von der anderen Richtung kommend ergab eine systematische Literaturrecherche [28] wenige Ausführungen über Geschlechterunterschiede in verschiedenen gesundheitsrelevanten Bereichen bei Kindern, wie z. B. Motorik und Bewegung [3, 5, 14] oder Körperbild [47, 49]. Diese Unterschiede werden zwar benannt, aber nicht ausreichend erklärt, als dass daraus theoriegestützte Interventionsansätze abgeleitet werden können [28]. Es sind v. a. Theorien zur Entwicklung und Stärkung des Kindes mit einem Fokus auf das Verhalten zu finden, die Berücksichtigung der Genderperspektive bleibt außen vor.

Eine systematische Praxisrecherche [28] schließlich ergab einige vielversprechende Ansätze zur Umsetzung geschlechtersensibler Prävention und Gesundheitsförderung für Kinder, die auf wenige Bundesländer verteilt sind. Diese Angebote und Maßnahmen wurden jedoch meist aus dem Bedarf heraus und häufig ohne theoretische Konzeption entwickelt. Dabei gibt es einerseits geschlechtersensible Angebote in Einrichtungen, die sich an alle Kinder richten (z. B. [51]). Andererseits gibt es Angebote, die grundsätzlich geschlechtshomogen arbeiten (z. B. [19]). In den meisten Angeboten werden Beratungen, Kurse, Gruppenstunden oder Workshops angeboten. Thematisch sind diese sehr breit gefächert von verschiedenen Präventionsthemen wie Gesundheitsförderung, Suchtprävention oder Prävention von (sexueller) Gewalt über Selbststärkung, Selbstbehauptung oder Selbstverteidigung bis hin zur körperlichen Entwicklung, Ernährung oder Bewegung (z. B. [4, 21]).

Insgesamt fehlen also konzeptionell-theoretische und praktisch fundierte Konzepte und Handlungsempfehlungen, wie geschlechtersensible Prävention bei Kindern in der Praxis aussehen kann [32]. Aufgrund dieser Forschungslücken sollen im Rahmen der vorliegenden Arbeit die folgenden Fragen beantwortet werden.

1. Was empfehlen Wissenschaftler/innen bzgl. der geschlechtersensiblen Prävention und Gesundheitsförderung für Kinder in den verschiedenen Settings und bzgl. der Qualifizierung entsprechender Praktiker/innen?
2. Wie beurteilen Praktiker/innen die Empfehlungen der Wissenschaftler/innen zur geschlechtersensiblen Prävention und Gesundheitsförderung für Kinder und welche Chancen und Herausforderungen sind dabei zu berücksichtigen?
3. Welche Empfehlungen lassen sich ableiten, um Kommunen und Anbietende bei der Entwicklung geschlechtersensibler präventiver und gesundheitsförderlicher Maßnahmen und Interventionen für Kinder zu unterstützen?

## Methoden

Die Forschungsfragen wurden im Rahmen des Projekts „Geschlechtersensible Prävention und Gesundheitsförderung: von der Beschreibung zur Intervention“ (PrevBoGi) in einem mehrschrittigen Verfahren bearbeitet. Dabei wurden qualitative Forschungsansätze genutzt, um die Erfahrungs- und Wissensbestände von Expertinnen und Experten aus Wissenschaft und Praxis sowie deren interprofessionellen Austausch explorativ erfassen zu können. Für dieses Vorgehen wurde bei der Ethikkommission der Medizinischen Fakultät der Heinrich-Heine-Universität Düsseldorf ein Ethikvotum eingeholt (Studiennummer 2019-388).

### Datenerhebung und -auswertung

#### Frage 1.

Zunächst wurde ein Workshop mit 12 Wissenschaftler/innen durchgeführt. Die Rekrutierung erfolgte in einem mehrstufigen Verfahren. In einer ersten Literaturrecherche wurden bundesweit und im deutschsprachigen Ausland (Österreich und Schweiz) Personen aus den für die Thematik relevanten Bereichen Geschlechterforschung, Entwicklungspsychologie, Erziehungswissenschaften, Jugendhilfe, Public Health und Medizin identifiziert. Im weiteren Verlauf wurden mithilfe des Schneeballverfahrens weitere Wissenschaftler/innen rekrutiert [28]. Der explorative Workshop war angelehnt an die Methode der nominalen Gruppentechnik [50]. Diese Methode sieht mehrere Phasen (Einzelarbeit, Diskussionen etc.) vor und eignet sich zur Ideenentwicklung und deren Priorisierung. Als Stimulus wurden Ergebnisse der vorgelagerten systematischen Literaturrecherche und einer empirischen geschlechterdifferenzierten Analyse zur Präventionsnutzung präsentiert [28]. Auf Basis der Stimulusfrage (Was empfehlen Sie bzgl. der geschlechtersensiblen Prävention und Gesundheitsförderung in den verschiedenen Settings und der Qualifizierung entsprechender Praktiker/innen?) folgte die stille Ideenfindung. Im Anschluss wurden in mehreren Diskussionsrunden erste Ideen zur geschlechtersensiblen Prävention zunächst gesammelt, vorgestellt und in der Gruppe diskutiert. Anschließend wurden gemeinsam Empfehlungen formuliert und diese nach Relevanz in einem Ranking bewertet. Ziel war die gemeinsame Priorisierung und Bewertung. Die Empfehlungen und entsprechenden Dimensionen dienten als Raster für die zweite Frage.

#### Frage 2.

Im zweiten Schritt wurden jeweils drei qualitative Fokusgruppen mit Fachkräften in Düsseldorf (*n* = 7, *n* = 6, *n* = 8) und Köln (*n* = 6, *n* = 8, *n* = 4) durchgeführt. Die Praktiker/innen wurden aus verschiedenen Bereichen der kommunalen Prävention rekrutiert, v. a. aus den Bereichen Kita, Schule oder mit Schwerpunkt in der kindheitsbezogenen Ernährung, Bewegung, Sprachförderung, Kinder- und Jugendarbeit oder therapeutischen Beratung. Ergänzend wurden Fachkräfte aus den verschiedenen Netzwerken von Multiplikatoren in den beiden Großstädten, die den Kooperationsverbund getragen haben (Düsseldorf und Köln) sowohl per E‑Mail als auch telefonisch rekrutiert.

Die moderierten Diskussionen fanden im Frühjahr 2020 statt. Die ersten Empfehlungen auf Basis des Expertenworkshops (vgl. Tab. 1) wurden dabei bewusst als kurzer inhaltlicher Stimulus genutzt. Es folgte eine Diskussion zur geschlechtersensiblen Prävention und Gesundheitsförderung auf Basis eines Leitfadens. Die Fokusgruppen wurden aufgezeichnet, extern wortgetreu transkribiert und von 3 Gutachterinnen mithilfe der Software MaxQDA (Verbi GmbH, Berlin, Deutschland) in Anlehnung an die inhaltlich-strukturierende qualitative Inhaltsanalyse nach Kuckartz [31] ausgewertet. Von allen Teilnehmenden liegen schriftliche Einwilligungserklärungen für die Aufzeichnung und Verarbeitung vor. Bei dem iterativen Vorgehen leiteten sich aus dem Stimulus (den Dimensionen aus Frage 1) jeweils fünf Oberkategorien ab. Im Auswertungsprozess wurden eine zusätzliche Oberkategorie sowie Unterkategorien induktiv gebildet. Im gegenseitigen Austausch wurde ein einheitliches Kategoriensystem entwickelt und die Kategorien zur Prüfung der Relevanz nach Häufigkeit der Nennung und Thematisierung in den Gruppendiskussionen gewichtet. Zu den finalen Oberkategorien „Übergeordnete und gesamtgesellschaftliche Themen im Kontext der geschlechtersensiblen Prävention und Gesundheitsförderung“, „Ressourcen“, „Setting/Lebenswelt“, „Qualifizierung“, „Partizipation“ und „Individuum“ wurden thematische Zusammenfassungen erstellt [31, 36].

#### Frage 3.

An dem finalen Online-Workshop zur Erstellung der Handlungsempfehlungen nahmen 12 Personen aus Wissenschaft und Praxis teil, die bereits in den vorangegangenen Befragungen beteiligt waren. Diese erhielten vorab eine Zusammenfassung der bisherigen Erkenntnisse in Berichtsform. Bei dem Workshop wurde diese Zusammenfassung zunächst kurz präsentiert. Im Anschluss wurden die Ergebnisse und verschiedene Schwerpunkte anhand von strukturierenden Leitfragen diskutiert. Basierend auf dieser Diskussion wurden die finalen Empfehlungen, die Kommunen und Anbietenden bei der Entwicklung geschlechtersensibler präventiver und gesundheitsförderlicher Maßnahmen und Interventionen unterstützen sollen, in einem letzten Schritt gemeinsam erarbeitet und in einer Synopse für die Fachöffentlichkeit zusammengefasst.

## Ergebnisse

Nachfolgend werden die Ergebnisse einzeln zur jeweiligen Fragestellung dargestellt.

### Frage 1.

Was empfehlen Wissenschaftler/innen bzgl. der geschlechtersensiblen Prävention in den verschiedenen Settings und der Qualifizierung entsprechender Praktiker/innen?

Die fünf wichtigsten Dimensionen, die zuvor im Workshop priorisiert worden waren, wurden im Nachgang zu ersten Empfehlungen der geschlechtersensiblen Prävention ausformuliert und von den strukturellen Faktoren (Ressourcen) über die Interaktion betreffende Faktoren (Partizipation) hin zu den das Individuum betreffende Faktoren (Geschlecht) angeordnet (Tab. 1).

**Tab. 1:** Ergebnisse aus dem Expertenworkshop mit Wissenschaftler/innen

Dimension	Empfehlung
Ressourcen	Es müssen Ressourcen (rechtliche, ökonomische, personelle und kompetenzbasierte) zur Verfügung stehen
Setting	Das Thema der Geschlechtersensibilität muss als Querschnittsthema im Setting verankert werden (Dokumente, Sprache, Stellenbesetzung)
Qualifizierung	Beteiligte Akteure und Fachkräfte müssen in der Thematik der Geschlechtersensibilität befähigt werden (Fortbildung, Selbstreflexion)
Partizipation	Kinder, Eltern, Fachkräfte und weitere kommunale Akteure sollten an der Entwicklung geschlechtersensibler Präventionsangebote beteiligt sein
Geschlecht	Mit verschiedenen Methoden kann „Geschlecht“ mit den Kindern thematisiert werden. Gleichzeitig sollte „Geschlecht“ wieder aus dem Fokus genommen werden

### Frage 2.

Wie beurteilen Praktiker/innen die Empfehlungen der Wissenschaftler/innen zur geschlechtersensiblen Prävention, und welche Chancen und Herausforderungen sind dabei zu berücksichtigen?

Die Ergebnisse aus den Fokusgruppen zu den oben dargestellten Dimensionen und ersten Empfehlungen sind nachfolgend in Form von thematischen Zusammenfassungen dargestellt [31, 36]. Die Dimensionen wurden im Prozess ergänzt und modifiziert.

### Übergeordnete und gesamtgesellschaftliche Themen im Kontext der geschlechtersensiblen Prävention und Gesundheitsförderung

Ein zentraler Punkt der Fachkräftediskussion war die Berücksichtigung des familiären Kontextes, der Lebenslage oder des Sozialraums bei der geschlechtersensiblen Förderung von Kindern. Das Kind entwickelte sich abhängig von diesen und weiteren übergeordneten Einflüssen wie bspw. gesellschaftlichen oder kulturellen Werten und Normen oder medialen Inhalten. Die Befragten diskutierten, dass die Gesellschaft bestimme, was „Normalität“ in Bezug auf das Geschlecht bedeutet, und wie wichtig eine Loslösung von diesen Bewertungen sei. Eine Vielfalt in Kultur, Geschlecht, Lebenswelten etc. und deren Akzeptanz und Toleranz sei, laut den Fachkräften, nur als gesamtgesellschaftliche Aufgabe zu lösen.

Geschlechterspezifische Zuschreibungen, Stereotypisierungen oder Rollenvorstellungen seien tief verwurzelt und über Generationen in der Gesellschaft verankert. Die Fachkräfte beschrieben eine Diskrepanz zwischen in der Familie, in der Einrichtung und der Gesellschaft vermittelten Werten als problematisch. Laut den Aussagen würden große Unterschiede bei Werten oder Rollenvorstellungen zwischen städtischen und ländlichen Regionen, Nationalitäten, Religionen etc. herrschen, welche Auswirkungen auf das Kind haben können.

### Ressourcen

Die Fachkräfte seien generell bei ihrer Arbeit mit Kindern, je nach Einrichtungsart, an rechtliche Grundlagen und Rahmenbedingungen auf Bundes- und Landesebene gebunden. Jedoch fehle es an einheitlichen Regelungen in der Art und Umsetzung von präventiven Maßnahmen. Viele Einrichtungen entschieden selbst über Art und Umfang der Maßnahmen in Abhängigkeit von den begrenzten finanziellen und personellen Ressourcen. Daher sei es häufig schwierig über die festgelegten Rahmenbedingungen hinaus zu agieren.

Die Stellenbesetzung oder Weiterbildungsmöglichkeiten von Fachkräften würden generell maßgeblich von finanziellen Ressourcen abhängen. Diese sahen sich mit einer zunehmenden Verantwortung in ihren Aufgabenbereichen konfrontiert und wünschten sich eine bessere Verteilung der finanziellen Ressourcen. So erscheine es sinnvoll, dass Kommunen unterstützend bei der Umsetzung von Prävention wirken. Prävention sollte als Bildungsaspekt berücksichtigt werden und Maßnahmen sollten nicht nur für vulnerable Gruppen zur Verfügung stehen, sondern alle Kinder einschließen. Es wird eine paritätische Stellenbesetzung angestrebt, jedoch sei dies aufgrund des Fachkräftemangels nur schwer umsetzbar. Besonders in Kitas und im Vorschul- und Grundschulbereich seien männliche Fachkräfte stark unterrepräsentiert.

Um einen rechtlichen Rahmen für die Handlungsfähigkeit im Umgang mit den Kindern zu schaffen und die Persönlichkeitsrechte der Kinder und Fachkräfte zu schützen, hielten die Befragten ein Schutzkonzept in jeder Institution im Kinder- und Jugendbereich für wichtig und sinnvoll.

### Setting/Lebenswelt

Kinder würden zu Hause bzw. in der Familie und in den Einrichtungen häufig mit unterschiedlichen Wertvorstellungen oder Rollenbildern konfrontiert. Auf eine wertschätzende Art und Weise sollte in den Einrichtungen darüber eine Auseinandersetzung stattfinden und es sollten verschiedene Vorstellungen zusammengebracht und gegenseitiger Respekt erarbeitet werden. Kinder sollten die Unterschiede nicht als Kluft begreifen, sondern verschiedene Lebenswelten, Rollenbilder etc. kennenlernen.

In Bezug auf die Stellenbesetzung scheine es sinnvoll, Fachkräfte mit Personalverantwortung für Vielfalt und Diversität zu sensibilisieren. Denn es sei wichtig, bei der Stellenbesetzung Vielfalt in Geschlecht, Herkunft etc. zu repräsentieren. Wie bei den Ressourcen bereits thematisiert wurde, sei die paritätische Besetzung allerdings häufig schwierig in der Umsetzung.

Das Thema Geschlechtersensibilität sollte in den Einrichtungen nicht unbedingt Schwerpunktthema sein, sondern eher als Querschnittsaufgabe verstanden werden, um dies in allen Arbeitsfeldern zu implementieren und grundsätzlich mitzudenken.

Austausch, Vernetzung und gegenseitige Stärkung vereinfachten viele Prozesse, auch in Bezug auf die Geschlechtersensibilität. Laut Aussagen der Befragten funktioniere dies in Form von Steuerungsgruppen oder Arbeitskreisen besonders gut.

### Qualifizierung

Zunächst waren das Bewusstsein und die Reflexion über die persönliche Einstellung zu dem Thema Geschlecht und die eigenen Werte wichtig für die Befragten. In der Berufsausbildung werden i. Allg. Kompetenzen erworben, die grundlegend für die Entwicklung einer Grundhaltung seien. Diese sollte wertfrei und neutral sein und von einer allgemeinen Gleichberechtigung der Kinder ausgehen, welche allerdings in der Praxis häufig schwer umzusetzen sei. Man sollte daher versuchen, nah und lebensweltorientiert mit einer akzeptierenden Haltung auf Augenhöhe und subjektorientiert mit den Familien zu arbeiten.

Fort- und Weiterbildungen seien ebenfalls sehr wichtig, es herrschten jedoch unterschiedliche Meinungen dazu, ob das Angebot ausreiche. Allerdings bestand Einigkeit, dass es im Berufsalltag selten möglich sei, entsprechende Angebote wahrzunehmen. Deshalb sollten die Bereiche Reflexion, Kultursensibilität und Geschlechtersensibilität verstärkt in Form von Pflichtmodulen in Ausbildung und Studium thematisiert werden. Es sei eine Reform nötig, um mit den neuen Herausforderungen des gesellschaftlichen Wandels umzugehen.

### Partizipation

Generell sei es wichtig, alle Beteiligten bei der Entwicklung und Durchführung von Angeboten zu berücksichtigen. Partizipation muss lebenswelt- und subjektorientiert stattfinden. Die Möglichkeiten der Partizipation hingen jedoch von der Motivation der Familien ab. Man sollte auf diese zugehen, sie mitnehmen und ihre Sichtweisen und Sorgen ernst nehmen, ihnen aber mit einer eigenen bzw. neutralen Haltung begegnen.

Für Kinder sei ein Rahmen, aber auch Grenzen der Partizipation wichtig. Ihre Ansichten würden in den Einrichtungen berücksichtigt, sie können sich einbringen, mitentscheiden oder Wünsche äußern. In einigen Einrichtungen sei die Partizipation von Eltern und Kindern fester Bestandteil in Form von Versammlungen, Räten etc.

### Individuum

Kinder lernten durch Nachahmen und orientierten sich an Vorbildern, Regeln und Gruppen. Daher sollte für die Kinder eine wohlfühlende und sichere Umgebung geschaffen werden, in der sie sich an verschiedenen Vorbildern orientieren können und lernen, anderen Haltungen und Werten mit Respekt und Toleranz zu begegnen. Eine vertrauensvolle Basis zwischen Fachkräften und Kindern sei wichtiger Bestandteil der Bildungsarbeit.

Fachkräfte sollten das Aufbrechen von Geschlechterkategorien fördern. Zum einen sei es wichtig, den Kindern die Möglichkeit zur freien und geschlechterneutralen Entfaltung zu geben. Andererseits wurde betont, dass es auch wichtig ist, Themen wie das biologische Geschlecht bewusst aufzugreifen und sich mit den Kindern in einer nicht belehrenden und stereotypisierenden Form mit dem Verständnis von Geschlecht auseinanderzusetzen. Aktivitäten sollten geschlechterneutral und für alle Kinder zugänglich sein. Das bedeutet nicht, dass Jungen und Mädchen sich nicht für geschlechtstypische Aktivitäten entscheiden dürfen, jedoch sollte ihnen eine freie und geschlechterunabhängige Wahl ermöglicht werden. Kinder sollten ebenso in ihrer Persönlichkeit und ihrer Selbstwirksamkeit gestärkt werden, um zu starken und selbstbewussten Individuen heranwachsen zu können.

#### Frage 3.

Welche Empfehlungen lassen sich ableiten, um Kommunen und Anbietende bei der Entwicklung geschlechtersensiblen präventiven und gesundheitsförderlichen Maßnahmen und Interventionen zu unterstützen?

In der Infobox 1 sind die ausgewählten Handlungsempfehlungen zur Umsetzung geschlechtersensibler Prävention und Gesundheitsförderung dargestellt, welche in dem finalen Workshop mit Expertinnen und Experten aus Wissenschaft und Praxis gemeinsam erarbeitet wurden.

#### Infobox 1 Handlungsempfehlungen zur Umsetzung geschlechtersensibler Prävention und Gesundheitsförderung

- Bei Prävention und Gesundheitsförderung das Geschlecht des Kindes im Zusammenhang mit seiner Lebenslage adressieren.
- Gelegenheiten für Kinder und Eltern schaffen, um verschiedene Geschlechterrollen und Lebensweisen zu thematisieren und kennenzulernen.
- Qualitätssicherung in Settings und bei Angeboten beachten, wie z. B. die partizipative Gestaltung, um eine freie Entwicklung bzw. Entfaltung zu ermöglichen.
- Sichtbarmachung in der Dokumentation von Praxisprojekten in Hinblick auf Geschlechtersensibilität bei Institutionen und Organisationen.
- Diversität in professionellen Teams berücksichtigen.
- Gezielte und bedarfsgerechte Einbindung des Themas in die relevanten Aus- und Fortbildungsgänge und Qualifikationsmaßnahmen.
- Bedarfsgerechter Austausch zum Thema Geschlechtersensibilität und möglichen Einflussfaktoren in professionellen und sozialen Netzwerken in Kommunen und Quartieren sowie deren Distribution.

## Diskussion

Ziel dieser Arbeit war es, praktische Handlungsempfehlungen für geschlechtersensible Prävention für Jungen und Mädchen zu entwickeln. Dabei wurden qualitative Forschungsansätze in 3 Schritten genutzt, um die Erfahrungs- und Wissensbestände von Expertinnen und Experten aus Wissenschaft (FF1) und Praxis (FF2) sowie deren interprofessionellen Austausch (FF3) explorativ erfassen zu können. Die resultierenden sieben Empfehlungen werden nachfolgend diskutiert und eingeordnet.

### Geschlecht und Lebenslage adressieren

Zunächst wurde die Relevanz von Kultursensibilität und familiärer sozialer Lage deutlich. In den Gruppendiskussionen wurde aufgegriffen, was bereits seit geraumer Zeit in der Ungleichheitsforschung gefordert wird: eine Loslösung von einzelnen Attributen oder Kategorien [45]. In der Geschlechterforschung wird dieser Aspekt unter dem Begriff der Intersektionalität untersucht. Darunter wird verstanden, dass soziale Kategorien wie Geschlecht, Ethnizität, Nationalität etc. nicht getrennt bzw. isoliert voneinander betrachtet werden können, sondern im Zusammenspiel berücksichtigt werden müssen [39]. Dass sich die soziale Lage je nach Geschlecht unterschiedlich auf Gesundheitsdeterminanten auswirkt, zeigt sich empirisch beispielsweise in der KiGGS-Studie: Demnach ist der soziale Gradient des Konsums zuckerhaltiger Getränke bei Jungen deutlich steiler als bei Mädchen. Und während der Alkoholkonsum bei Jungen mit der sozialen Lage steigt, findet sich bei Mädchen kein systematischer Zusammenhang [33]; bzgl. der Präventionsnutzung zeigen neuere Analysen, dass die soziale Lage von Jungen und Mädchen eine größere Rolle spielt als das Geschlecht [52]. Bereits bestehende Konzepte der geschlechtersensiblen Prävention verweisen ebenfalls auf die Relevanz der Lebenswelten in Zusammenhang mit dem Geschlecht und fordern eine Ausdifferenzierung klar umrissener Subzielgruppen (z. B. [2]).

### Geschlechterrollen und Lebensweisen kennenlernen

Um den Kindern die Möglichkeit zu geben, sich auszuprobieren und sich frei zu entwickeln, können bspw. die Methoden der *Dramatisierung* und *Entdramatisierung* aus der geschlechtsbezogenen Pädagogik genutzt werden [17]. Dabei wird das Geschlecht explizit zum Thema gemacht (dramatisiert), z. B. in Form von geschlechterhomogenen Sport- und Bewegungsgruppen. In einem weiteren Schritt soll dann sichtbar und erfahrbar sein, dass das Geschlecht nur eines von vielen Unterscheidungsmerkmalen ist. Solche entdramatisierenden Herangehensweisen sollen den paradoxen Effekt verhindern, dass durch Dramatisierung Geschlechterstereotypen befördert werden. So kann man z. B. besonders in geschlechterhomogenen Gruppen die Unterschiede innerhalb der Gruppe herausarbeiten. Eine *Nicht-Dramatisierung* ermöglicht darüber hinaus die individuelle Förderung der Kinder in verschiedenen Bereichen ohne Bezug zur Kategorie Geschlecht, z. B. durch Kompetenztrainings [12]. Diese Methoden sind u. E. wichtige Ergänzungen bestehender Konzepte der geschlechtersensiblen Prävention und sie können in verschiedenen Settings und Maßnahmen für Jungen und Mädchen angewendet werden. Hierbei ist jedoch zu berücksichtigen, dass für Trans*- und Inter*-Kinder Homogenitätsannahmen und Gruppenteilungen diskriminierend wirken können [13]. Als Kompromiss könnten Trans*- und Inter*-Kinder sich bei Gruppentrennung derjenigen Geschlechtergruppen zuordnen, die sie situativ als passend empfinden. Dieser Aspekt wurde allerdings von den teilnehmenden Expertinnen und Experten nicht thematisiert.

### Partizipative Gestaltung

Partizipation, d. h. Beteiligung der Zielgruppen, wird seit jeher als Qualitätskriterium von Prävention und Gesundheitsförderung herausgestellt [27], so auch bei der geschlechtersensiblen Prävention. Die Kriterien der Assessmentqualität [44] setzen sich beispielsweise explizit mit der Frage auseinander, ob die Bedürfnisse der anvisierten Zielgruppen erfasst wurden. Konzeptionell veranschaulichen Partizipationsleitern [54] verschiedene Beteiligungsmöglichkeiten von Mitbestimmung bis Entscheidungsmacht. Bei den identifizierten Praxisbeispielen zur geschlechtersensiblen Prävention werden partizipatorische Elemente explizit benannt, wie der Einsatz von Kinderversammlungen, Kinderräten etc., bei denen die Kinder eigene Ideen einbringen oder aktiv mitentscheiden können (z. B. [4, 19, 41]). Partizipative Gestaltung als Qualitätssicherung geht jedoch noch weiter. Sie bedeutet nicht nur eine möglichst starke Teilhabe der Zielgruppe (hier: der Kinder), sondern auch der Projektmitarbeiter/innen und weiteren relevanten Akteuren an den verschiedenen Entwicklungsphasen von Maßnahmen einschließlich Bedarfsbestimmung, Interventionsplanung, Umsetzung und Evaluation [54]. Partizipative Qualitätsentwicklung basiert auf dem lokalen Wissen der Beteiligten. Klassische Elemente der Arbeit beinhalten den ständigen Austausch mit Adressaten und innerhalb der Teams [51], Selbstevaluation und Reflexion [15]. Einige Praxisbeispiele veröffentlichen hierzu regelmäßig Ergebnisberichte [25], vereinzelt sind wissenschaftlich begleitete Evaluationen zu finden [4].

### Dokumentation von Praxisprojekten

Es lassen sich aktuell nur wenige explizit geschlechtersensible Praxisprojekte der Prävention und Gesundheitsförderung für Kinder ausfindig machen [29]. Zu begründen ist dies einerseits durch das fehlende Angebot von Projekten und Programmen, andererseits durch die mangelhafte Dokumentation. Östlin et al. [42] haben den Mangel an Informationen zu geschlechtersensiblen Interventionen bereits vor geraumer Zeit kritisiert und eine bessere Dokumentation und Dissemination gefordert. In einer jüngeren Studie wurde von Fachkräften moniert, dass allgemein viele Förderangebote für Kinder existieren, diese jedoch nicht schriftlich ausformuliert sind und auch keine Internetpräsenz besteht, z. B. in gängigen Praxisdatenbanken [30]. Dieser Punkt ist in der Debatte um geschlechtersensible Prävention recht neu und es ist zu hoffen, dass die Digitalisierung der Angebotslandschaft durch die COVID-19-Pandemie („coronavirus disease 2019“) Aufwind erfährt [18].

### Diversität in professionellen Teams

Die Geschlechterverteilung in Projektteams wurde in der Literatur zur geschlechtersensiblen Prävention bereits thematisiert [24, 44] und in der Altersgruppe der Kinder ist sie besonders bedeutsam. Der Anteil von männlichem pädagogischen Personal in Kindertageseinrichtungen für Kinder bis 6 Jahre lag 2019 bei 6,4 % [16], an den Grundschulen bei 15,7 % [48]. Zwar ist die Tendenz steigend, durch die Unterbesetzung von männlichen Fachkräften ist eine Diversität in den Teams jedoch kaum realisierbar, was als grundlegendes Strukturproblem zu verstehen ist und eine tatsächliche Geschlechtersensibilität verhindert. 62 % der Eltern und 94 % des pädagogischen Personals in Kitas fordern, dass sich die Politik für mehr männliche Erzieher einsetzt [53]. Trotz des großen Interesses mangelt es bislang an langfristigen und koordinierten Strategien zur Erhöhung des Männeranteils [40]. Ein wichtiger Schritt ist beispielsweise die Einrichtung der Koordinationsstelle *Männer in Kitas*, welche den Anteil männlicher Fachkräfte in Kindertagesstätten mittel- und langfristig zu steigern versucht [53]. Die Gründe für dieses Ungleichgewicht sind vielfältig, wie z. B. die niedrige Entlohnung, geringe Aufstiegschancen, befristete Arbeitsverhältnisse oder das verbreitete stereotype Berufsbild [9, 53]. Außerdem sind Männer allein aufgrund der Geschlechtszugehörigkeit bei dem Thema Missbrauch einem höheren Verdächtigungsrisiko ausgesetzt als Frauen. In einer Befragung gaben 32 % aller Eltern an, an die Gefahr eines möglichen Missbrauchs durch männliche Erzieher gedacht zu haben, bei Erzieherinnen sind es 13 % [53]. Um diesem *Generalverdacht* zu begegnen, ist ein professioneller Umgang, beispielsweise in Form von Schutzkonzepten, unerlässlich [11].

### Geschlechtersensibilität in Qualifikationsmaßnahmen

Der Ansatz der geschlechtergerechten Strukturqualität [44] hat die Frage nach der Qualifikation des Personals zuvor schon aufgeworfen. Und zumindest für die Multiplikator/innen männerspezifischer Gesundheitsproblematiken wurden zu wenige Qualifizierungsangebote moniert [2]. Die aktuellen Fokusgruppen zur Zielgruppe der Kinder kamen zu keinem klaren Ergebnis, inwiefern Geschlechtersensibilität ausreichend in Ausbildung, Studium sowie Fort- und Weiterbildung berücksichtigt wird. Die objektive Betrachtung zeigt: Die Bildungspläne zur Ausbildung der Fachkräfte liegen im Aufgabenbereich der Länder und deren Ausgestaltung obliegt meist den einzelnen (Fach)schulen. Beim Vergleich der Lehrpläne fällt auf, dass z. B. Schleswig-Holstein und Thüringen die Beschlüsse der Kultusministerkonferenzen weitestgehend übernommen haben. Baden-Württemberg hat ein eigenes Lernfeld *Gender-Mainstreaming* in seinen Lehrplänen vorgesehen und NRW hat den Bereich der sogenannten *Genderkompetenz* in die bestehende Lernfelder integriert [10, 38]. Auf Ebene der Fachschulen sowie vielen universitären Studiengängen, z. B. Sozialpädagogik, ist Genderkompetenz vorrangig als Querschnittsthema in andere übergeordnete Module integriert. Dies erfolgt im Rahmen der allgemeinen Gesetzesvorgaben, die Art und Weise der thematischen Umsetzung ist jedoch schwer nachvollziehbar und wenig vergleichbar. Module und Veranstaltungen, die Genderkompetenz explizit benennen, finden sich häufig im Wahlpflichtbereich [10]. Darüber hinaus werden viele Weiterbildungsmöglichkeiten für Fachkräfte zur allgemeinen Genderkompetenz angeboten, wenige Beispiele finden sich für die Kombination aus Gender-Mainstreaming und Gesundheitsförderung [1, 23, 34].

### Austausch und Vernetzung

Der Gedanke der Vernetzung in Sachen Geschlecht und Gesundheit ist nicht neu. Östlin et al. [42] haben bereits auf die überregionalen Akteure wie die Weltgesundheitsorganisation oder die Vereinten Nationen hingewiesen, die mit ihren Rahmenkonzepten oder Entwicklungszielen eine Grundlage für gemeinsames Engagement bilden. Für die Zielgruppe der Kinder sind regionale Netzwerke hilfreich wie etwa die Landesarbeitsgemeinschaften zur Jungen- oder Mädchenarbeit, die es in den meisten Bundesländern gibt (z. B. [35, 37]). Daran anknüpfend finden regelmäßig thematische Fachtagungen oder -foren beispielsweise von der Bundesarbeitsgemeinschaft Jungenarbeit [6] statt.

### Stärken und Limitationen der Studie

Unseres Wissens nach ist dies eine der ersten Studien, die sich mit der praktischen Umsetzung von geschlechtersensibler Prävention und Gesundheitsförderung explizit für die Zielgruppe der Kinder auseinandersetzt. Dabei wurden Aspekte aus der bestehenden Literatur bestätigt, aber es ergaben sich auch neue Punkte. Eine Stärke der Studie liegt in der Berücksichtigung mehrerer Perspektiven. Hierzu wurden die Expertise auf wissenschaftlich-theoretischer Ebene aus verschiedenen Fachdisziplinen (Public Health, Erziehungs- und Sportwissenschaften etc.) und die Expertise auf praktischer Ebene, bestehend aus Professionen und Personen, die in der Prävention mit Jungen und Mädchen arbeiten, gebündelt. Durch die Verknüpfung verschiedener qualitativer Methoden wurden die Ergebnisse stetig weiterentwickelt. Eine zentrale Einschränkung der Fokusgruppen ist die Zusammensetzung der Stichprobe. Männliche Fachkräfte waren in den Diskussionen deutlich unterrepräsentiert. Dieser Umstand ist vermutlich dadurch zu erklären, dass der frühkindliche Bereich durch weibliche Fachkräfte dominiert wird, wie bereits oben beschrieben. Eine Einschränkung ist die Fokussierung unserer Studie auf Geschlecht als binäre Kategorie. Geschlechtliche Vielfalt im Kindesalter wird auch in der Fachöffentlichkeit immer sichtbarer, beispielsweise in der Kinder- und Jugendhilfe [20]. Damit ergeben sich für die geschlechtersensible Prävention für Kinder künftig neue Herausforderungen, die auch von der Forschung aufgegriffen werden sollten.

## Fazit für die Praxis

- Geschlechtersensible Prävention für Jungen und Mädchen betrifft viele Ebenen – von politischen und gesellschaftlichen Maßnahmen bis hin zur individuellen Arbeit mit dem Kind durch qualifiziertes Personal. Diesbezüglich gibt es noch viel zu tun, insbesondere was die Darstellung und Evaluation von Leuchtturm-Projekten betrifft.
- Vor dem Hintergrund der pandemischen Entwicklungen hat die gesundheitliche Förderung von Jungen und Mädchen eine noch größere Relevanz erreicht, wobei sich die Frage der zielgruppensensiblen Ansätze einmal mehr stellt.

## Supplementary Information

Kategoriensystem

## References

[1] Akademie für Kindergarten, Kita und Hort (2020) Fortbildungen 2020/2021. Lehrgänge, Seminare, Teamfortbildungen, Fernkurse. Für Kita-Leitungen, Erzieher, Fachkräfte und pädagogische Mitarbeiter. https://www.kindergartenakademie.de/new/zip/Seminarprogramm.pdf. Zugegriffen: 30. Nov. 2020

[2] Altgeld T (2007) Warum weder Hänschen noch Hans viel über Gesundheit lernen – Geschlechtsspezifische Barrieren der Gesundheitsförderung und Prävention. Präv Gesundheitsf 2(2):90–97

[3] Ardelt-Gattinger E, Ring-Dimitriou S, Hofmann J et al (2016) Geschlechtsunterschiede bei psychologischen, ernährungs- und sportwissenschaftlichen Einflussfaktoren auf Adipositas/Übergewicht bei Kindern und Jugendlichen in Österreich (Gender differences of psychological, nutritional, and physical fitness variables influencing obesity/overweight in Austrian children and adolescents). Wien Med Wochenschr 166(3–4):111–116 26847439 10.1007/s10354-015-0427-9

[4] Armut und Gesundheit in Deutschland e. V. (2016) Street Jumper. https://www.gesundheitliche-chancengleichheit.de/praxisdatenbank/street-jumper/. Zugegriffen: 13. Dez. 2022

[5] Boxberger K, Reimers AK (2019) Parental correlates of outdoor play in boys and girls aged 0 to 12‑A systematic review. IJERPH. 10.3390/ijerph16020190 10.3390/ijerph16020190 PMC6351982 30641874

[6] Bundesarbeitsgemeinschaft Jungenarbeit e. V. (2021) Fachforum. www.bag-jungenarbeit.de. Zugegriffen: 13. Dez. 2022

[7] Bundesvereinigung Prävention und Gesundheitsförderung (2013) Prinzipien guter Prävention und Gesundheitsförderung. Leitbild der Bundesvereinigung Prävention und Gesundheitsförderung e. V. (BVPG). verabschiedet am 09.04.2013 auf der Mitgliederversammlung, Berlin, Bonn

[8] Bundeszentrale für gesundheitliche Aufklärung (2009) Gender Mainstreaming in der Gesundheitsförderung/Prävention. Anwendungsorientierter Austausch zwischen Forschung und Facharbeit unter besonderer Berücksichtigung der Anforderungen an eine geschlechtersensible Gesundheitsförderung bei sozial Benachteiligten. Dokumentation des BZgA-Workshops, Köln, 18. Apr. 2008

[9] Collenberg F (2020) Männliche Erzieher als Vorbilder im Kindergarten. Geschlechter- und rollentheoretische Aspekte. Bachelorarbeit, Universität Siegen

[10] Cremers M, Krabel J (2012) Gender macht Schule – wie viel Gender steckt in der Fachschulausbildung für Erzieher/innen? In: Cremers M, Höyng S, Krabel J et al (Hrsg) Männer in Kitas. Barbara Budrich, S 183–198

[11] Cremers M, Krabel J (2012) Generalverdacht und sexueller Missbrauch in Kitas: Bestandsanalyse und Bausteine für ein Schutzkonzept. In: Cremers M, Höyng S, Krabel J et al (Hrsg) Männer in Kitas. Barbara Budrich, S 265–285

[12] Debus K (2012) Geschlechterreflektierte Arbeit mit Jungen an der Schule. Texte zu Pädagogik und Fortbildung rund um Jungenarbeit, Geschlecht und Bildung. Dissens e.V, Berlin

[13] Debus K (2017) Dramatisierung, Entdramatisierung und Nicht-Dramatisierung von Geschlecht und sexueller Orientierung in der geschlechterreflektierten Bildung. (Wie) Kann ich geschlechterreflektiert arbeiten, ohne Stereotype zu verstärken? In: Glockentöger I, Adelt E (Hrsg) Gendersensible Bildung und Erziehung in der Schule, 1. Aufl. Grundlagen – Handlungsfelder – Praxis. Waxmann, Münster

[14] Demetriou Y, Vondung C, Bucksch J et al (2019) Interventions on children’s and adolescents’ physical activity and sedentary behaviour: protocol for a systematic review from a sex/gender perspective. Syst Rev 8(1):65 30808402 10.1186/s13643-019-0963-2 PMC6390303

[15] Der Kinderschutzbund. Kreisverband Nürnberg (2019) Achtung Grenze! https://www.kinderschutzbund-nuernberg.de/angebote/fuer-einrichtungen/achtung-grenze/. Zugegriffen: 13. Dez. 2022

[16] Destatis (2019) Pressemitteilung Nr. N 009 vom 18. November 2019. Männerberufe, Frauenberufe? Klassische Rollenbilder bestimmen noch immer die Arbeitswelt. https://www.destatis.de/DE/Presse/Pressemitteilungen/2019/11/PD19_N009_122.html. Zugegriffen: 13. Dez. 2022

[17] Faulstich-Wieland H (1996) Abschied von der Koedukation? In: Kleinau E, Opitz C (Hrsg) Geschichte der Mädchen- und Frauenbildung. Campus, Frankfurt/New York, S 386–400

[18] Franz S, Simon-Kutscher K, Kunze S et al (2022) Familiales Hilfesuchverhalten während der COVID-19-Pandemie und die Verschiebung in den digitalen Raum. Sucht 68(1):19–27

[19] Frauen- und Mädchengesundheitszentrum MEDEA e. V. (2021) Mädchenprojekt Maxi. https://medea-dresden.de/maedchenprojekt-maxi/. Zugegriffen: 13. Dez. 2022

[20] Der Paritätische Gesamtverband (2021) Geschlechtliche Vielfalt in der Kinder- und Jugendhilfe inter* und trans*Kinder 0–6 Jahre, Berlin

[21] GesundheitsLaden e. V. (2021) Jungen im Blick. Eine Beratungs- und Präventionsstelle für Jungen* und junge Männer* in Stuttgart. https://www.jungen-im-blick.de. Zugegriffen: 13. Dez. 2022

[22] Inchley J, Currie D, Young T (Hrsg) (2016) Growing up unequal. Gender and socioeconomic differences in young people’s health and well-being; Health Behaviour in School-Aged Children (HBSC) Study: international report from the 2013/2014 survey. Health policy for children and adolescents, No. 7.. World Health Organization, Regional Office for Europe, Copenhagen

[23] Institut für den Situationsansatz (ISTA) (2020) Sie kommen zu uns – Fortbildungsprogramm 2020. https://situationsansatz.de/fortbildung/fortbildungen-im-ista/. Zugegriffen: 30. Nov. 2020

[24] Jahn I, Kolip P (2002) Die Kategorie Geschlecht als Kriterium für die Projektförderung von Gesundheitsförderung Schweiz

[25] Kindertreffpunkt e. V. (2021) Kindertreffpunkt Oskar-Maria-Graf-Zentrum. https://www.gesundheitliche-chancengleichheit.de/praxisdatenbank/kindertreffpunkt-oskar-maria-graf-zentrum/. Zugegriffen: 13. Dez. 2022

[26] Kolip P (2008) Geschlechtergerechte Gesundheitsförderung und Prävention (Gender sensitive health promotion and prevention). Bundesgesundheitsblatt Gesundheitsforschung Gesundheitsschutz 51(1):28–35 18185966 10.1007/s00103-008-0416-x

[27] Kooperationsverbund Gesundheitliche Chancengleichheit (2017) Kriterien für gute Praxis der soziallagenbezogenen Gesundheitsförderung. Kooperationsverbund Gesundheitliche Chancengleichheit, Köln, Berlin

[28] Kreffter K, Nguyen TH, Wessely S et al (2021) Geschlechtersensible Prävention und Gesundheitsförderung: Von der Beschreibung zur Intervention (PrevBoGi). Ergebnis-Zusammenfassung. Zenodo

[29] Kreffter K, Nguyen TH, Wessely S et al (2021) Geschlechtersensible Prävention und Gesundheitsförderung: Von der Beschreibung zur Intervention (PrevBoGi). Ergebnis-Zusammenfassung. Zenodo. 10.5281/zenodo.7043396

[30] Kreffter K, Wahl S, Dragano N et al (2019) Familien mit Bedarf sind Familien, auf die wir zugehen müssen. Eine partizipative Bedarfsanalyse zur kommunalen Prävention für sozioökonomisch benachteiligte Kinder. Präv Gesundheitsf 25(3):263

[31] Kuckartz U (2016) Qualitative Inhaltsanalyse. Methoden, Praxis, Computerunterstützung, 3. Aufl. Grundlagentexte Methoden. Beltz Juventa, Weinheim, Basel

[32] Kuger S, Kluczniok K, Sechtig J et al (2011) Gender im Kindergarten. Empirische Datenlage zu Unterschieden zwischen Mädchen und Jungen. Z Padagog 57(2):269–288

[33] Kuntz B, Waldhauer J, Zeiher J et al (2018) Soziale Unterschiede im Gesundheitsverhalten von Kindern und Jugendlichen in Deutschland – Querschnittergebnisse aus KiGGS Welle 2. RKI-Bib1 (Robert Koch-Institut)

[34] LAG Jungenarbeit NRW (2020) Hör’ mal wer da hämmert. Konzepte, Tools und Methoden für die Jungenarbeit. https://lagjungenarbeit.de/veranstaltungen/hoer-mal-wer-da-haemmert. Zugegriffen: 30. Nov. 2020

[35] LAG Mädchenpolitik Bayern e. V. (2021) Mädchenarbeit in Bayern. http://lag-maedchenpolitik-bayern.de/. Zugegriffen: 13. Dez. 2022

[36] Lamnek S (2010) Qualitative Sozialforschung. Lehrbuch, 5.. Aufl. Grundlagen Psychologie. Beltz, Weinheim (Online-Materialien)

[37] Landesarbeitsgemeinschaft Jungen- und Männerarbeit Sachsen e. V. (2021) Landesfachstelle Jungenarbeit. https://www.juma-sachsen.de/. Zugegriffen: 13. Dez. 2022

[38] Ledig M (2011) Fort- und Weiterbildung von Lehrkräften an Fachschulen für Sozialpädagogik. Ergebnisse einer Interviewstudie mit Schulleitungen, Stand: September 2011. WiFF-Studien, 12: Ausbildung. Deutsches Jugendinstitut, München

[39] Lenz I (2010) Intersektionalität. In: Becker R, Kortendiek B (Hrsg) Handbuch Frauen- und Geschlechterforschung. VS, Wiesbaden, S 158–165

[40] Katholische Hochschule für Sozialwesen, Sinus Sociovision (2010) Männliche Fachkräfte in Kindertagesstätten. Eine Studie zur Situation von Männern in Kindertagesstätten und in der Ausbildung zum Erzieher. Sinus Sociovision, Katholische Hochschule für Sozialwesen, Berlin (Forschungsprojekt)

[41] MiM – Mädchen in Marzahn e. V. (2021) Seminare und Workshops für Mädchen und Schule. https://www.mim-ev.de/bildung-und-beratung/bildungsprojekte.html. Zugegriffen: 13. Dez. 2022

[42] Östlin P, Eckermann E, Mishra US et al (2006) Gender and health promotion: a multisectoral policy approach. Health Promot Int 21(1):25–35 17307954 10.1093/heapro/dal048

[43] Ravens-Sieberer U, Wille N, Erhart M et al (2008) Prevalence of mental health problems among children and adolescents in Germany. Results of the BELLA study within the National Health Interview and Examination Survey. Eur Child Adolesc Psychiatry 17(1):22–33 19132301 10.1007/s00787-008-1003-2

[44] Ruckstuhl B, Kolip P, Gutzwiller F (2001) Qualitätsparameter in der Prävention. In: Salice-Stephan K (Hrsg) Qualitätsmanagement in Gesundheitsförderung und Prävention. Grundsätze, Methoden und Anforderungen ; eine aktuelle Bestandsaufnahme, 1. Aufl. Bundeszentrale für Gesundheitliche Aufklärung, Köln, S 38–50

[45] Scherr A (2014) Diskriminierung als Kategorie der Ungleichheitsforschung. In: Scherr A (Hrsg) Diskriminierung und soziale Ungleichheiten. Springer, Wiesbaden, S 5–9

[46] Schmidt SC, Schneider J, Reimers AK et al (2019) Exploratory determined correlates of physical activity in children and adolescents: the MoMo study. IJERPH. 10.3390/ijerph16030415 10.3390/ijerph16030415 PMC6388266 30709045

[47] Simen-Kapeu A, Veugelers PJ (2010) Should public health interventions aimed at reducing childhood overweight and obesity be gender-focused? BMC Public Health 10:340 20546619 10.1186/1471-2458-10-340 PMC2894777

[48] Statistisches Bundesamt (2020) Pressemitteilung Nr. N 034 vom 3. Juli 2020. Lehrkräfte an allgemeinbildenden Schulen: 2018/19 war mehr als ein Drittel 50 Jahre und älter. Statistisches Bundesamt, Wiesbaden

[49] Tatangelo GL, Ricciardelli LA (2013) A qualitative study of preadolescent boys’ and girls’ body image: gendered ideals and sociocultural influences. Body Image 10(4):591–598 24018337 10.1016/j.bodyim.2013.07.006

[50] van de Ven AH, Delbecq AL (1972) The nominal group as a research instrument for exploratory health studies. Am J Public Health 62(3):337–342 5011164 10.2105/ajph.62.3.337 PMC1530096

[51] Verbund sozialpädagogischer Projekte e. V. (2021) Kinder‑, Jugend- und Familienhaus „Plauener Bahnhof“. https://www.vsp-dresden.org/familie-freizeit/plauener-bahnhof/uebersicht.html. Zugegriffen: 13. Dez. 2022

[52] Weyers S, Kreffter K, Götz S et al (2022) Are we promoting boys and girls equally? An analysis of boys’ and girls’ participation in community prevention. J Public Health. 10.1007/s10389-022-01750-y

[53] Wippermann C (2018) Kitas im Aufbruch – Männer in Kitas. Die Rolle von Kitas aus Sicht von Eltern und pädagogischen Fachkräften. DELTA-Institut für Sozial- und Ökologieforschung, Berlin (Sozialwissenschaftliche Repräsentativbefragung)

[54] Wright MT, Kilian H, Block M et al (2015) Partizipative Qualitätsentwicklung: Zielgruppen in alle Phasen der Projektgestaltung einbeziehen (Participatory Quality Development: Engaging Community Members in All Phases of Project Planning and Implementation). Gesundheitswesen 77(Suppl 1):S141–S142 24937351 10.1055/s-0033-1347268

