# Supplementary material for: Gender-sensitive prevention and health promotion for children: opportunities and challenges in practicability
Source: Pravent Gesundh. 2022 Dec 29:1–9. [Article in German] Online ahead of print. doi: 10.1007/s11553-022-01006-3 (PMC9799705; doi:10.1007/s11553-022-01006-3)
Supplement: Supplementary file 1 — Kategoriensystem [file 11553_2022_1006_MOESM1_ESM.pdf]

Oberkategorie	Subkategorie 1. Ebene	Subkategorie 2. Ebene
Modell allgemein	Modellverständnis / Umsetzbarkeit	
	Verbesserungsvorschläge am Modell	
	gesellschaftlicher Kontext	gesellschaftliche Entwicklung des Rollenbildes
		gesellschaftliche Entwicklung von Werten und Normen
		fehlende Anerkennung für soziale Berufe
		Wirtschaftszweig Geschlechtertypisierung
	kultureller / religiöser Kontext	
	Sprache	
	systemischer Ansatz	
Ressourcen	rechtliche / politische Rahmenbedingungen	Schutzkonzepte
	finanzielle Ressourcen	
	personelle Ressourcen	
	kompetenzbasierte Ressourcen	
Setting	Diskrepanzen zwischen Rollenbildern verschiedener Settings	
	Vielfalt in der Stellenbesetzung	
	Settingübergreifende Zusammenarbeit	
	Querschnittsthema	
Qualifizierung	Haltung	
	Vorbildfunktion	
	(Selbst-) Reflexion	
	Elternarbeit	
	Ausbildung / Schulung / Fortbildung	
Partizipation	Art und Weise	Eltern
		Kinder
	Bezugspersonen-Vielfalt	
Individuum	Methoden	Lernen durch Nachahmen
		Selbsterfahrung
		Persönlichkeitsstärkung
		Bücher und (Lehr-) Materialien
	Konzepte / Ansätze	Lebenslaufperspektive
		Dramatisierung und Entdramatisierung
		individuelle Bedürfnisse
		weitere Konzepte / Maßnahmen / Programme
